# Caffeic acid phenethyl ester promotes haematopoietic stem/progenitor cell homing and engraftment

**DOI:** 10.1186/s13287-017-0708-x

**Published:** 2017-11-07

**Authors:** Xiaofang Chen, Yi Han, Bowen Zhang, Yiming Liu, Sihan Wang, Tuling Liao, Ziliang Deng, Zeng Fan, Jing Zhang, Lijuan He, Wen Yue, Yanhua Li, Xuetao Pei

**Affiliations:** 1 School of Laboratory Medicine and Biotechnology, Southern Medical University, No. 1838 Guangzhou Avenue North, Baiyun District, Guangzhou, 510515 China; 2 Stem Cell and Regenerative Medicine Lab, Beijing Institute of Transfusion Medicine, No. 27 Taiping Road, Haidian District, Beijing, 100850 China; 3 South China Institute of Biomedicine, No. 1 Luoxuan 4th Road, Haizhu District, Guangzhou, 510005 China; 4 Guangdong Medical University, No. 1 Xincheng Road, Dongguan, 523808 China

**Keywords:** Caffeic acid phenethyl ester, Haematopoietic stem/progenitor cells, Homing, Engraftment

## Abstract

**Background:**

Several studies have suggested that caffeic acid phenethyl ester (CAPE) can induce the expression of hypoxia inducible factor-1α (HIF-1α) protein. We determined whether CAPE has a novel function in improving the homing and engraftment of haematopoietic stem/progenitor cells (HSPCs) by regulating HIF-1α gene expression in the bone marrow (BM) niche.

**Methods:**

For survival experiments, lethally irradiated C57BL/6 mice were injected with a low number of BM mononuclear cells (MNCs) and CAPE according to the indicated schedule. Homing efficiency analysis was conducted using flow cytometry and colony-forming unit (CFU) assays. The influence of intraperitoneal injection of CAPE on short-term and long-term engraftment of HSPCs was evaluated using competitive and non-competitive mouse transplantation models. To investigate the mechanism by which CAPE enhanced HSPC homing, we performed these experiments including Q-PCR, western blot, immunohistochemistry and CFU assays after *in-vivo* HIF-1α activity blockade.

**Results:**

CAPE injection significantly increased the survival rate of recipient mice after lethal irradiation and transplantation of a low number of BM MNCs. Using HSPC homing assays, we found that CAPE notably increased donor HSPC homing to recipient BM. The subsequent short-term and long-term engraftment of transplanted HSPCs was also improved by the optimal schedule of CAPE administration. Mechanistically, we found that CAPE upregulated the expression of HIF-1α, vascular endothelial growth factor-A (VEGF-A) and stromal cell-derived factor 1α (SDF-1α). The HIF-1α inhibitor PX-478 blocked CAPE-enhanced HSPC homing, which supported the idea that HIF-1α is a key target of CAPE.

**Conclusions:**

Our results showed that CAPE administration facilitated HSPC homing and engraftment, and this effect was primarily dependent on HIF-1α activation and upregulation of SDF-1α and VEGF-A expression in the BM niche.

**Electronic supplementary material:**

The online version of this article (doi:10.1186/s13287-017-0708-x) contains supplementary material, which is available to authorized users.

## Background

Haematopoietic stem cell transplantation (HSCT) has been applied for treating malignant and non-malignant haematologic diseases in the clinic for over 50 years [1, 2]. However, the curative rate of this treatment, especially for cord blood (CB) HSCT, must be improved because of the low homing efficiency of haematopoietic stem and progenitor cells (HSPCs), delayed engraftment and the occurrence of graft versus host diseases. Efficient homing and engraftment of HSPCs are critical for haematopoietic repopulation in patients after transplantation, and these processes depend on several important steps. First, infused HSPCs find their way to bone marrow (BM) microvessels via rolling and firm adhesion to endothelial cells. Subsequently, the HSPCs migrate across the BM endothelial cells (BMECs) and extracellular matrix barrier and lodge in the BM niche. Complicated molecular interactions are involved in the homing process, including selection with its ligand, chemotactic SDF-1/CXCR4 axis and adherent VCAM-1/VLA4 axis and so on [3–7]. Finally, the transplanted HSPCs undergo proliferation and multilineage differentiation in their BM home and reconstitute the haematopoietic and immune systems in the recipients.

Improving HSPC homing to the BM is important for facilitating subsequent engraftment and haematopoietic repopulation, particularly when the number of infused HSPCs is limited [8]. Based on the understanding of the mechanism of HSPC homing, several approaches have been reported to promote the seeding of HSPCs to the BM and enhance engraftment. Ex-vivo manipulation of HSPCs through incubation with prostaglandin E2 (PGE2), BMP4 or fucosyltransferase-VI can accelerate HSPC homing and engraftment in the BM [9–12]. Accumulated evidence shows that the chemoattractant factor SDF-1α plays a crucial role in HSPC homing and subsequent engraftment [13–19]. Although several agents regulate HSPC seeding to the BM and enhance their engraftment, encouraging more HSPCs to find their way to the BM is still a big challenge.

Caffeic acid phenethyl ester (CAPE) is a propolis extract obtained from honeybee hives and an ester analogue of caffeic acid. Increasing evidence indicates that CAPE has multiple beneficial properties, such as antibacterial, anti-viral, anti-inflammatory and anti-cancer properties [20–25]. Previous studies have suggested that CAPE can induce the expression of hypoxia inducible factor-1α (HIF-1α) protein, which occurred by inhibition of HIF prolyl hydroxylase, the key enzyme for von Hippel–Lindau-dependent HIF-1α degradation, and activate the expression of HIF-1α target genes, such as vascular endothelial growth factor (VEGF) and haeme oxygenase-1 (HO-1), which play protective roles in ischemia/reperfusion injury [26–28]. In our previous study, we found that CAPE upregulated the expression of HIF-1α and stem cell factor (SCF) to promote the expansion of HSPCs in vitro [29]. Since HIF-1α, an upstream transcriptional factor, regulates the expression of SDF-1α and because SDF-1α is a critical chemotactic factor for HSPC migration, we speculate that CAPE may enhance HSPC homing to the BM.

Herein, we show that CAPE administration to irradiated recipients promotes the recruitment and homing of HSPCs to the BM niche in a BM transplantation model. The subsequent enhanced short-term and long-term engraftment of transplanted HSPCs was also improved by the optimal schedule of CAPE administration. This occurs primarily via HIF-1α activation and by affecting the expression of SDF-1α and VEGF in the BM niche. Thus, we present a novel role for CAPE: improving the homing and engraftment of HSPCs.

## Methods

### Mice

C57BL/6-CD45.2 mice (CD45.2 mice, 6–8 weeks old, male, 20–24 g) were purchased from Beijing Vital River Laboratory Animal Technology Company, Ltd. C57BL/6-CD45.1 mice (CD45.1 mice, 6–8 weeks old, male, 20–24 g) were purchased from the Institute of Haematology, Chinese Academy of Medical Sciences (Tianjin, China). All animal experiments were reviewed and approved by the Animal Center Committee of the Academy of Military Medical Sciences (Beijing, China). After lethal irradiation, mice were fed with distilled water containing gentamicin.

### Radiation and treatment

Mice were lethally irradiated (950 cGy, 80–120 cGy/min) using a ^60^Co irradiator. CAPE (Sigma) concentrated solution was prepared in saline containing 5% dimethyl sulphoxide (DMSO; Sigma), 20% propanediol and 0.2% Tween 80. Mice were injected intraperitoneally with CAPE daily at the indicated doses for 3 days—that is, day –1 (24 h before irradiation), day 0 (0 h after irradiation) and day +1 (24 h after irradiation)—while the control mice were injected intraperitoneally with the equal volume of vehicle used for CAPE dissolution. The HIF-1α inhibitor PX-478 (Xcess Biosciences) was dissolved in DMSO at a concentration of 20 μg/μl. Mice were injected intraperitoneally with 5 mg/kg PX-478 in PBS just after daily CAPE injections.

### Transplantation experiments

For survival experiments, lethally irradiated mice were injected with 2.5 × 10^5^ bone marrow mononuclear cells (BM MNCs) and CAPE according to the indicated schedule. For homing experiments, lethally irradiated CD45.2 mice were injected intraperitoneally with vehicle or 3.0 mg/kg CAPE for 3 days. Twenty hours after irradiation, these recipient mice were injected with 2 × 10^7^ BM MNCs from CD45.1 mice. For CFU-S12 detection, lethally irradiated mice were treated with vehicle or 3.0 mg/kg CAPE for 3 days and were injected with 1.5 × 10^5^ BM MNCs 20–24 h after irradiation. For the competitive transplantation experiment, BM MNCs (5 × 10^5^) were harvested from the primary recipient mice 20 h after bone marrow transplantation (BMT) and the various treatments, and were then mixed with 5 × 10^5^ competitor CD45.2^+^ BM MNCs and co-transplanted into irradiated CD45.2 mice (secondary recipients).

### Immunophenotype analysis using flow cytometry

BM samples were collected from the femurs and tibias of mice and then lysed with erythrocyte lysing buffer. The remaining cells were first stained with Fixable Viability Stain 510 (FVS510; BD Bioscience). For blood cell lineage detection, the cells were stained with lineage antibodies against CD3e, CD11b, Ter-119, B220, Gr-1 and matched isotype controls (eBioscience). To examine the percentages of LSK (Lin^–^Sca-1^+^c-Kit^+^) cells, BM cells were stained with biotin-labelled lineage antibodies and then incubated with streptavidin APC-eFluor® 780-labelled secondary antibody (eBioscience), along with BV605-labelled anti-mouse Sca-1 (BD Bioscience) and APC-labelled c-Kit (eBioscience). For the detection of engraftment and chimerism, the BM cells were stained with PE or FITC-labelled antibodies against CD45.1 or CD45.2 (eBioscience). All samples were detected by BD FACS Aria (BD Bioscience).

### Colony-forming unit assays

BM MNCs were collected and cultured in MethoCult™ GF M3434 medium (STEMCELL Technologies). Seven days later, typical colonies, including granulocyte–erythroblast–macrophage–megakaryocyte colony-forming units (CFU-GEMM), erythrocyte burst-forming units (BFU-E), granulocyte–macrophage colony-forming units (CFU-GM), megakaryocyte colony-forming units (CFU-Meg) and granulocyte colony-forming units (CFU-G), were visually scored based on morphological criteria using a light microscope.

### Cytokine array

BM samples were collected from lethally irradiated C57BL/6 mice 20 h after 2 × 10^7^ BM MNC transplantation with vehicle or 3.0 mg/kg CAPE injection from day –1 to day +1. The femurs of three mice per group were flushed using 5 ml PBS. The pooled supernatants per group were collected after centrifugation, freeze-dried and resuspended in 120 μl PBS. Cytokine array was performed using the RayBio® Mouse Cytokine Antibody Array G-Series 3 (RayBiotech) following the manufacturer’s instructions. Data analysis was performed using the analysis tool provided by the kit.

### BMEC culture and treatment with CAPE

BM MNCs were isolated from the femurs and tibias of mice. Primary mouse BMECs were obtained by culturing BM MNCs in EGM™-2-MV BulletKit™ medium (EGM2 medium, CC-3202; Lonza). Non-adherent cells were poured off 72 h after seeding cells in the medium. The BMECs were identified by immunofluorescence staining using antibodies against CD31 (Proteintech), VE-Cadherin (Abcam), Bs-lectin (Vector Laboratories) and Vwf (Proteintech). Primary BMECs were replated into new six-well plates with EGM2 medium. CAPE, dissolved in DMSO at 10 mg/ml, was added into the culture medium at different concentrations (0, 0.1 and 1.0 μg/ml). Cells were cultured at 37 °C in 5% CO_2_ for 24 h.

### Quantitative RT-PCR

Total RNA was obtained using TRIzol reagent (Invitrogen), and 1000 pg of RNA was reverse-transcribed into cDNA with ReverTra Ace qPCR RT Master Mix (Toyobo). All gene expression data were normalized by the housekeeping gene, β-ACTIN. Q-PCR analysis was performed with a Bio-Rad iQ5 system using Thunderbird SYBR qPCR Mix (Toyobo). The primer sequences are presented in Additional file 1: Table S1.

### Western blot analysis

The BMECs were resuspended in buffer containing proteinase inhibitors. The lysates were cleared by centrifugation. The concentration of the total protein per sample was detected using a BCA protein assay kit (Thermo Scientific) and 60 μg of protein sample was loaded for western blot analysis using antibodies against mouse β-ACTIN (1:2000; Cell Signaling Technology), HIF-1α (1:500; Novus Biologicals), SDF-1α (1:2000; Abcam) and VEGF-A (1:10,000; R&D Systems).

### Immunohistochemistry

The femurs and tibias of mice were dissected and fixed in 4% formalin. Bones were dehydrated after decalcification, embedded in paraffin and cut into sections. Immunostaining was performed according to the manufacturer’s recommendations (Vector Laboratories). BM sections were stained with primary antibodies, including monoclonal anti-mouse HIF-1α (1:20; Novus Biologicals), SDF-1α (1:200; Abcam) or VEGF-A (1:200; R&D Systems), and biotin-labelled secondary antibodies.

### Statistical analysis

Survival rate data were analysed using a log-rank test (GraphPad Prism 5; GraphPad Software). For comparison of two groups, data were analysed with two-tailed Student’s *t* tests, and *P* < 0.05 was significant. All error bar data represent the mean ± SD.

## Results

### CAPE increased the survival rate of mice after lethal irradiation and BMT

**Fig. 1:**
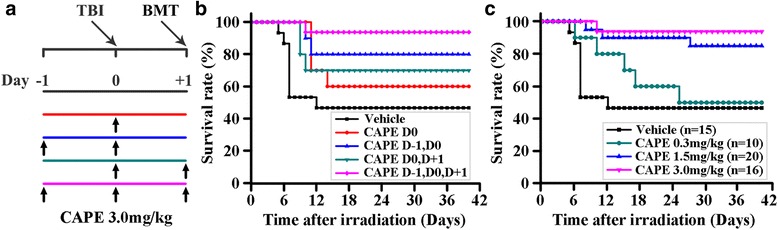
CAPE increased the survival rate. **a** BM transplantation and treatment model. All mice were lethally irradiated (950 cGy, 80–120 cGy/min) on day 0. Mice were injected intraperitoneally with 3.0 mg/kg of CAPE at different schedules. Each mouse was transplanted with 2.5 × 10^5^ BM MNCs on day +1. **b** Survival rates of the lethally irradiated recipients receiving BMT and CAPE injection with different schedules. All mice were administered vehicle or 3.0 mg/kg CAPE intraperitoneally. Vehicle: vehicle daily for 3 days (*n* = 15); CAPE D0: vehicle on day –1 and day +1, CAPE on day 0 (*n* = 10); CAPE D–1,D0: CAPE on day –1 and day 0, vehicle on day +1 (*n* = 10); CAPE D0,D + 1: vehicle on day –1, CAPE on day 0 and day +1 (*n* = 10); CAPE D–1,D0,D + 1: CAPE daily for 3 days (*n* = 16). **c** Survival rate of lethally irradiated mice receiving BMT and CAPE injection at different dosages. Lethally irradiated mice were injected with 0 (Vehicle), 0.3, 1.5 or 3.0 mg/kg CAPE from day –1 to day +1. TBI total body irradiation, BMT bone marrow transplantation, CAPE caffeic acid phenethyl ester

To determine whether CAPE treatment had a beneficial effect by regulating HSPC homing, we performed survival rate experiments on mice given CAPE injections. The mice received irradiation at day 0 and BMT at day +1. To determine an effective CAPE injection schedule, mice were administered 3.0 mg/kg of CAPE intraperitoneally at different schedules (Fig. 1a). The vehicle control group of mice with lethal irradiation and 2.5 × 10^5^ BM MNC transplantation had the lowest survival rate at 46.7% (Fig. 1b). The survival rates of the mice treated with CAPE for two doses (from day –1 to day 0 or from day 0 to day +1) were 70% and 80% separately, both of which were higher than that of the one dose of CAPE group (60%). We found that daily CAPE treatment for 3 days resulted in a 93.75% survival rate in mice with lethal irradiation and 2.5 × 10^5^ BM MNC transplantation (Fig. 1b), which indicated that three injections of CAPE were effective in elevating the survival rates of mice receiving BMT. To further determine the dose–response relationship between CAPE treatment and the improvements in the survival rate of mice receiving BMT, mice were injected with 0, 0.3, 1.5 or 3.0 mg/kg CAPE for 3 consecutive days. The 3.0 mg/kg CAPE dose was observed to be the optimal dose and resulted in the highest survival rate of the mice with BMT (Fig. 1c). These results indicated that administration of three doses of CAPE from day –1 to day +1 accelerated haematopoietic repopulation, which might primarily depend on the role of CAPE in regulating HSPC homing.

### CAPE promotes HSPC homing to the BM

**Fig. 2:**
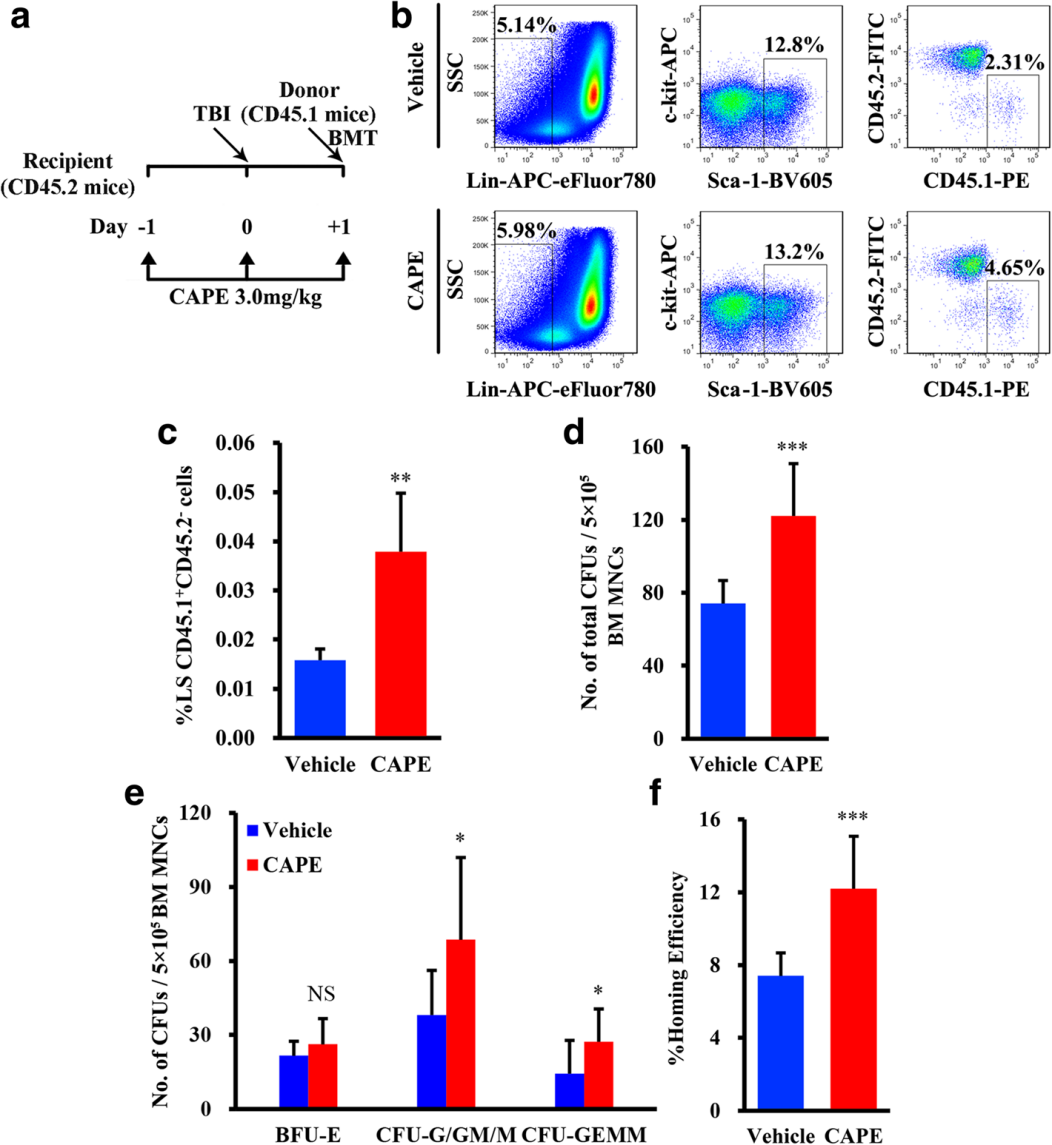
CAPE promoted HSPC homing to the BM niche. **a** Schematic representation of the homing experiments. Lethally irradiated CD45.2 mice were transplanted with 2 × 10^7^ CD45.1^+^ BM MNCs and injected with 3.0 mg/kg CAPE or vehicle daily for 3 days. **b** Representative dot plots for murine BM Lin^–^Sca-1^+^CD45.1^+^CD45.2^–^ cells. **c** Percentage of CD45.1^+^CD45.2^–^Lin^–^Sca-1^+^ cells in BM was increased by CAPE (*n* = 6). **d** Total CFU number generated from 5 × 10^5^ BM MNCs. BM MNCs were harvested for CFU assays 20 h after BMT (*n* = 5). **e** Number of different types of CFU (BFU-E, CFU-G/GM/M and CFU-GEMM) generated from 5 × 10^5^ BM MNCs. **f** Homing efficiency of CFUs calculated by comparing the homed CFU number with the initially injected CFU number. Blue indicates vehicle-treated group, red indicates CAPE-treated group; experiments repeated three times. Data presented as mean ± SD. *P < 0.05, **P < 0.01, ***P < 0.001, CAPE vs vehicle. TBI Total body irradiation, BM bone marrow, BMT bone marrow transplantation, CAPE caffeic acid phenethyl ester, SSC side scatter, CFU-G granulocyte colony-forming units, CFU-M macrophage colony-forming units, CFU-GEMM granulocyte–erythroblast–macrophage–megakaryocyte colony-forming units, CFU-GM granulocyte–macrophage colony-forming units, MNC mononuclear cell, NS not significant

To assess whether CAPE promoted HSPC homing to BM niches, lethally irradiated CD45.2 mice were injected intraperitoneally with three doses of vehicle or CAPE, and then BM MNCs isolated from CD45.1 mice were transplanted into the irradiated mice (Fig. 2a). Twenty hours later, BM cells from the recipients were harvested and analysed according to their cell surface marker expression. Using flow cytometry, we found that CAPE treatment led to a 2.4 ± 0.8-fold increase in the percentage of CD45.1^+^CD45.2^–^Lin^–^Sca-1^+^ cells in the BM of the recipients (*P* = 0.0037; Fig. 2b, c). We then used colony-forming unit (CFU) assays to further evaluate the ability of CAPE to regulate HSPC homing to the BM. Compared with the vehicle group, CAPE administration significantly increased the total number of CFUs in the BM of the recipients 20 h after BM transplantation (CAPE vs vehicle, 122 ± 28.7 vs 74 ± 12.7 CFUs per 5 × 10^5^ BM MNCs, *P* = 0.000057; Fig. 2d). We also analysed the number of different types of CFUs, including BFU-E, CFU-G/GM/M and CFU-GEMM, in the BM of the recipients. CAPE treatment caused significant increases in the number of CFU-G/GM/M and CFU-GEMM in the BM (*P* = 0.0143 and *P* = 0.0345; Fig. 2e). The BFU-E number between the two groups showed little increase. We then used a formula to calculate HSPC homing efficiency. The homing efficiency was determined by comparing the number of homed CFUs with the number of CFUs injected [30]. The results showed that the homing efficiency of HSPCs in the CAPE group significantly increased compared with the vehicle group (CAPE vs vehicle, 12.20 ± 2.87% vs 7.41 ± 1.27%, *P* = 0.000057; Fig. 2f).

### CAPE enhanced short-term engraftment of HSPCs in mice

**Fig. 3:**
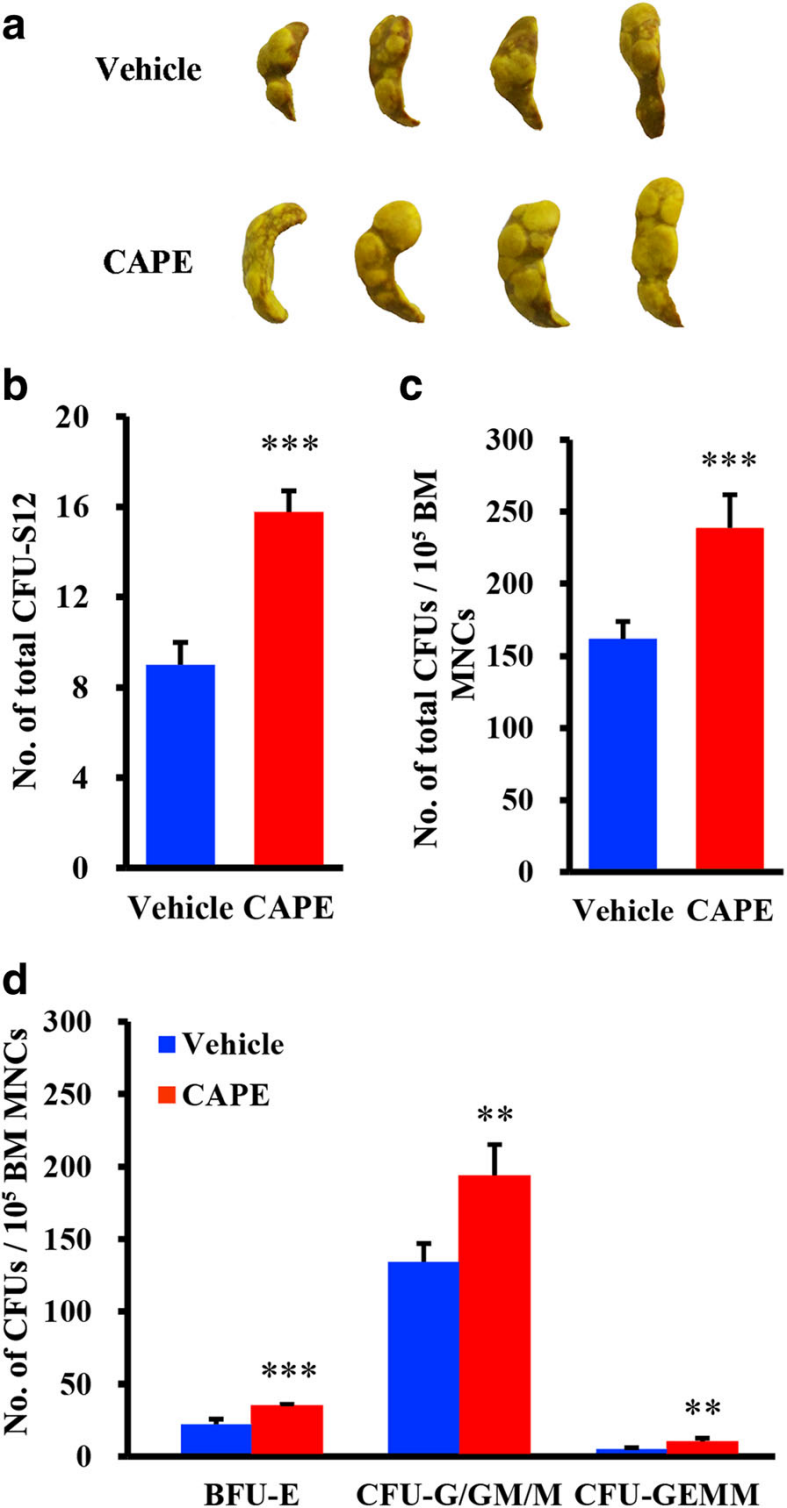
CAPE promoted short-term engraftment of HSPCs in the BM. **a** Morphology of CFU-S12. Lethally irradiated mice were treated with vehicle or 3.0 mg/kg CAPE from day –1 to day +1 and were injected with 1.5 × 10^5^ BM MNCs on day +1. Spleens were isolated from these mice on day 12 after BMT. **b** Total numbers of CFU-S12 counted. *n* = 8 in blue, *n* = 6 in red. **c**, **d** Total CFUs and different types of CFU numbers generated from 1 × 10^5^ BM MNCs (*n* = 5). BM MNCs harvested for CFU assays on day 12 after BMT. Blue indicates vehicle-treated group, red indicates CAPE-treated group; experiments repeated three times. Data presented as mean ± SD. **P < 0.01, ***P < 0.001, CAPE vs vehicle. BM bone marrow, CAPE caffeic acid phenethyl ester, CFU-G granulocyte colony-forming units, CFU-M macrophage colony-forming units, CFU-GEMM granulocyte–erythroblast–macrophage–megakaryocyte colony-forming units, CFU-GM granulocyte–macrophage colony-forming units, MNC mononuclear cell

We showed that CAPE promoted HSPC homing to the BM. To further determine whether increased seeding of HSPCs to the BM as a result of CAPE treatment would result in better short-term engraftment of transplanted HSPCs than vehicle treatment, we analysed the CFU-S12 and BM CFU numbers on day 12 after transplantation. We found that more CFU-S12 grew on the spleen in the CAPE group than in the vehicle group (CAPE vs vehicle, 15.75 ± 0.96 vs 9 ± 1.00 CFU-S, *P* = 0.000018; Fig. 3a, b). Similarly, an in-vitro CFU assay using BM cells harvested on day 12 after transplantation showed a significant increase in the total CFU number in the CAPE group (238.7 ± 23.12 CFUs per 1 × 10^5^ BM MNCs) compared with the vehicle group (161.7 ± 12.10 CFUs per 1 × 10^5^ BM MNCs, *P* = 0.0069; Fig. 3c). Consistently, the BM cells from the CAPE treatment group formed a larger number of different types of CFUs than those from the vehicle group (CAPE vs vehicle: for BFU-E, 35.0 ± 1.00 vs 22.3 ± 3.51, *P* = 0.0039; for CFU-G/GM/M, 193.7 ± 21.7 vs 134.3 ± 12.58, *P* = 0.0149; for CFU-GEMM, 10.0 ± 2.65 vs 5.0 ± 1.00 CFUs per 1 × 10^5^ BM MNCs, *P* = 0.0069; Fig. 3d). These results indicated that three doses of CAPE to the recipients significantly promoted short-term engraftment of HSPCs in mice.

### CAPE promoted long-term engraftment of HSPCs in mice

**Fig. 4:**
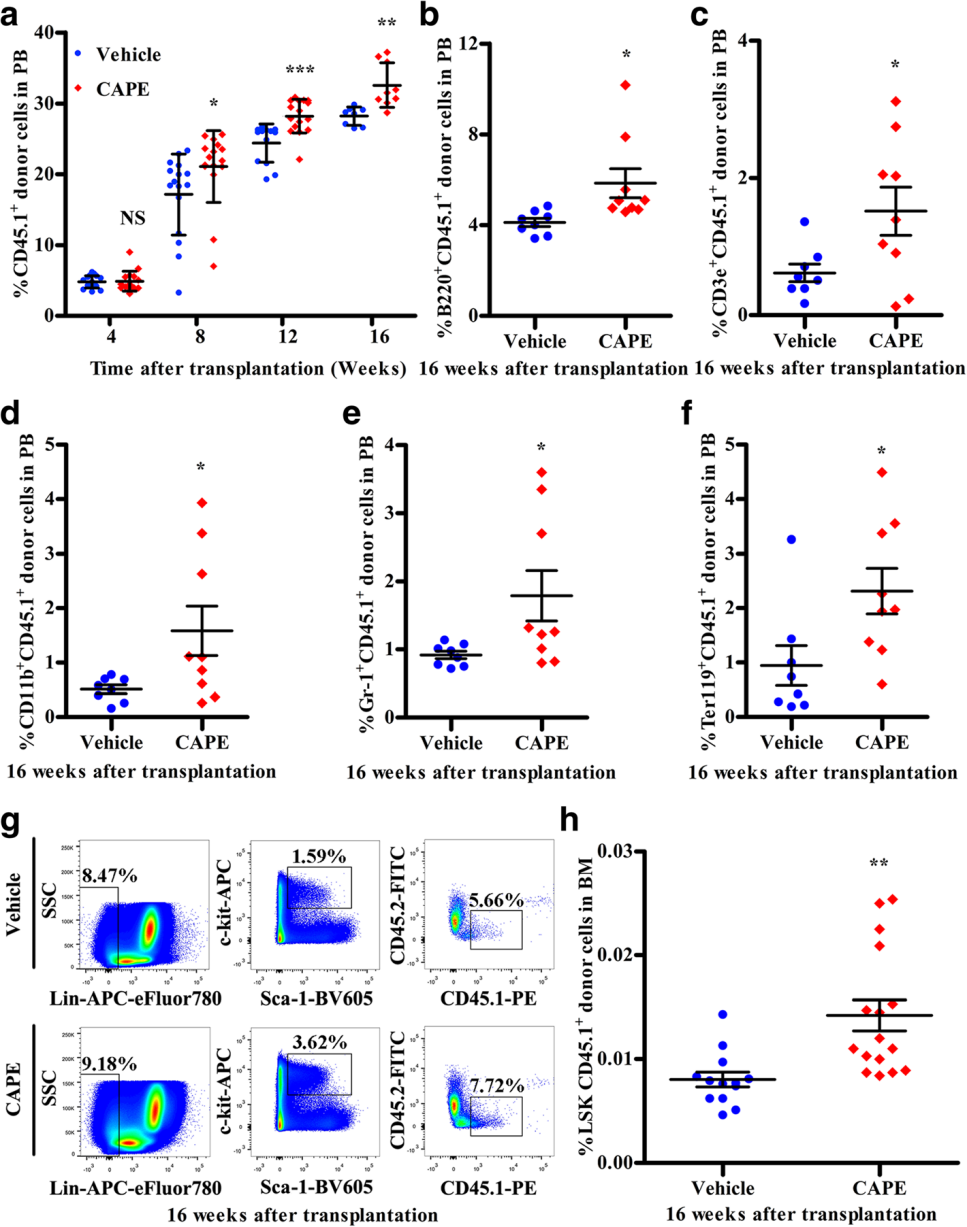
CAPE promoted long-term engraftment of HSPCs in the BM. **a** Percentages of CD45.1^+^ cells in PB every 4 weeks after secondary transplantation until the 16th week. BM MNCs (5 × 10^5^) were harvested from the primary recipient mice 20 h after BMT and the various treatments and were then mixed with 5 × 10^5^ competitor CD45.2^+^ BM MNCs and co-transplanted into irradiated CD45.2 mice (secondary recipients). Sixteen mice per group.**b**–**f** Donor CD45.1^+^ blood lineage cell percentages in the secondary recipient PB detected in the 16th week after secondary transplantation. *n* = 8 in blue, *n* = 9 in red. **g**, **h** Representative dot plots and percentage of BM Lin^–^Sca-1^+^c-kit^+^CD45.1^+^ cells from secondary recipients (*n* = 16). BM cells were harvested from the secondary recipient mice at the 16th week after competitive BMT. Blue indicates vehicle group, red indicates CAPE group. *P < 0.05, **P < 0.01, ***P < 0.001, CAPE vs vehicle. BM bone marrow, CAPE caffeic acid phenethyl ester, SSC side scatter, NS not significant, PB peripheral blood

We then applied competitive repopulation models to evaluate the influence of CAPE on long-term engraftment of HSPCs. The 5 × 10^5^ chimeric BM cells, obtained from CAPE-treated or vehicle-treated primary CD45.2 recipients 20 h following CD45.1^+^ BM cell transplantation, were transplanted into lethally irradiated secondary CD45.2 recipients. Then, 5 × 10^5^ BM cells from CD45.2 mice were co-transplanted into the lethally irradiated secondary CD45.2 recipients as competitor cells. Peripheral blood (PB) from secondary recipient mice was analysed for the presence of the primary donor (CD45.1) repopulation within 16 weeks after transplantation. The increase in the number of homed donor (CD45.1) haematopoietic cells in the mice that underwent CAPE administration was further revealed by increased engraftment of the primary donor CD45.1^+^ cells in the PB in the secondary recipients particularly at 8, 12 and 16 weeks after transplantation (Fig. 4a). We analysed the lineage percentage of primary CD45.1 donor blood cells in the secondary recipient PB. Mice transplanted with BM cells from CAPE-treated primary recipients had more CD45.1-derived B220^+^, CD3e^+^, CD11b^+^, Gr-1^+^, and Ter-119^+^ cells than mice that received cells from vehicle-treated mice (Fig. 4b–f). Four months after transplantation, long-term donor CD45.1^+^ LSK cell engraftment rates in the BM increased by approximately 1.77-fold in mice that received BM cells from the CAPE-treated primary recipient mice relative to mice that received cells from vehicle-treated primary recipients (Fig. 4g, h). These results strongly indicated that CAPE enhanced long-term engraftment of HSPCs, which was the result of CAPE-enhanced HSPC homing to the BM.

### CAPE upregulated the expression of HIF-1α, VEGF-A and SDF-1α

**Fig. 5:**
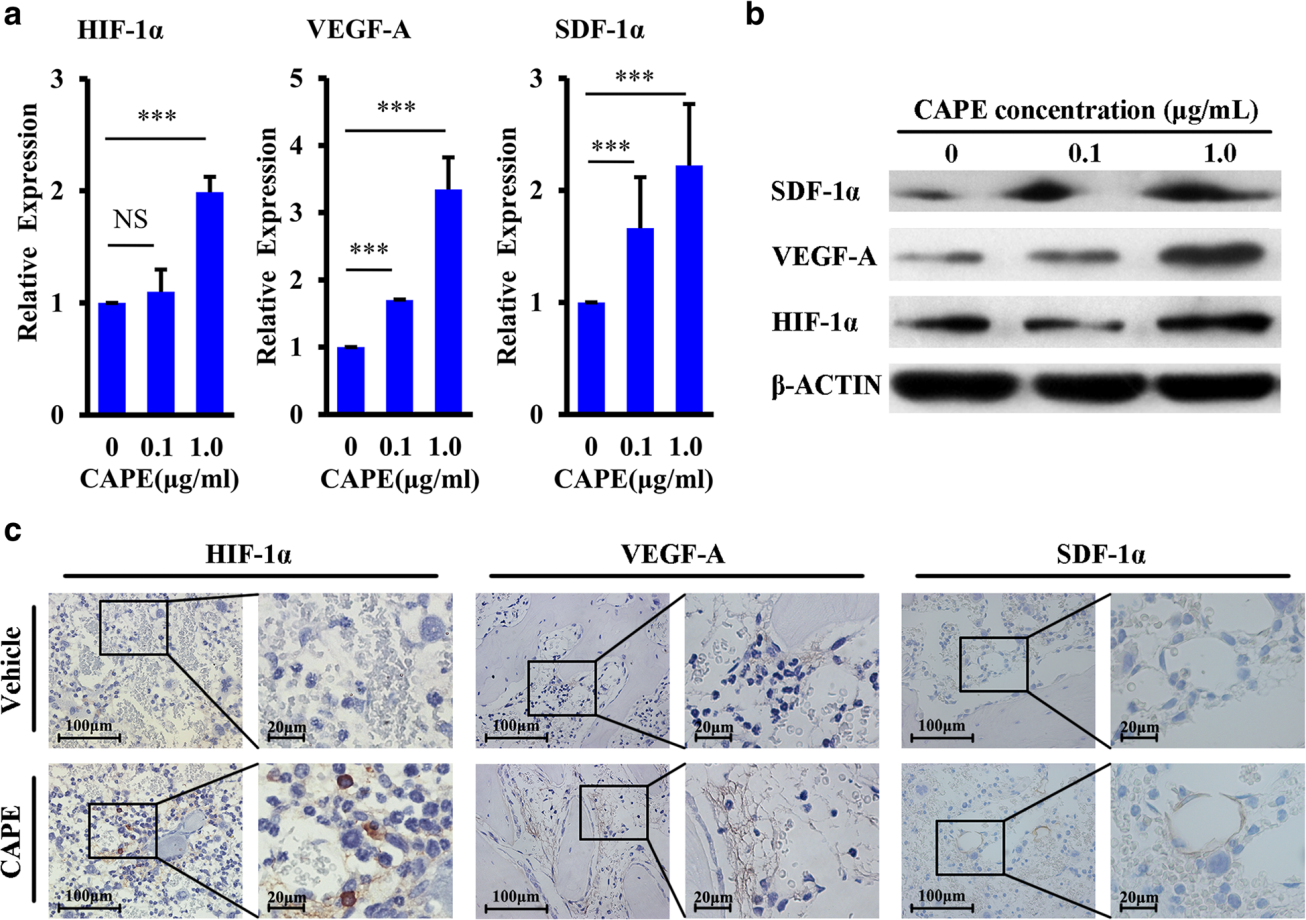
CAPE upregulated the expression of HIF-1α, VEGF-A and SDF-1α. **a** Gene expression levels of HIF-1α, VEGF-A and SDF-1α detected using Q-PCR. BMECs were incubated with CAPE at different concentrations (0, 0.1, 1.0 μg/ml) for 24 h. All gene expression data normalized by the housekeeping gene, β-ACTIN. ***P < 0.001, CAPE 1.0 μg/ml vs CAPE 0 μg/ml, CAPE 0.1 μg/ml vs CAPE 0 μg/ml. **b** Protein expression levels of HIF-1α, VEGF-A and SDF-1α in murine BMECs. BMECs were incubated with CAPE at different concentrations (0, 0.1, 1.0 μg/ml) for 24 h. Total protein of these cells was isolated and 60 μg protein per sample was loaded for western blot analysis. **c** Expression of HIF-1α, VEGF-A and SDF-1α proteins in the BM niche of lethally irradiated mice 20 h after BMT by IHC. Lethally irradiated mice were treated with vehicle or 3.0 mg/kg CAPE for three doses. All tests were repeated three times. CAPE caffeic acid phenethyl ester, HIF-1α hypoxia inducible factor-1α, NS not significant, SDF-1α stromal cell-derived factor 1α, VEGF vascular endothelium growth factor

To determine the mechanism by which CAPE promotes HSPC homing and engraftment, we harvested BM supernatants 20 h after transplantation from lethally irradiated mice treated with three doses of CAPE or vehicle. By applying a cytokine array, we found that the highest increases in VEGF-A occurred in the CAPE treatment group (Additional file 2: Figure S1). CAPE treatment also increased the levels of SDF-1α in the BM (Additional file 3: Figure S1). We then used Q-PCR and western blot analysis to assess whether the expression of VEGF-A and SDF-1α were activated in BM cells after CAPE treatment. Since HIF-1α is a target gene of CAPE [26, 29], we also detected the expression of HIF-1α in BMECs. The BMECs were incubated with CAPE at 0, 0.1 and 1.0 μg/ml for 24 h. The results showed that 1.0 μg/ml CAPE significantly upregulated the expression of HIF-1α, VEGF-A and SDF-1α genes and proteins in BMECs (Fig. 5a, b). BM stromal cells (Methods for isolation and characterization of BM stromal cells are showed in Additional file 3) showed little alteration in the expression level of these genes (Additional file 4: Figure S2), suggesting that CAPE acts on BMECs and increases the expression of HIF-1α, VEGF-A and SDF-1α. We then performed an immunostaining experiment to detect the expression of these proteins in the BM niche of lethally irradiated mice 20 h after transplantation. CAPE treatment led to enhanced expression of HIF-1α and SDF-1α proteins in BM cells and much stronger VEGF-A staining in BM stroma (Fig. 5c). These data suggested that CAPE-enhanced homing of HSPCs might be attributed to improvement of the BM niche by increasing SDF-1α and VEGF-A levels.

### HIF-1α inhibitor PX-478 blocked CAPE-enhanced HSPC homing

**Fig. 6:**
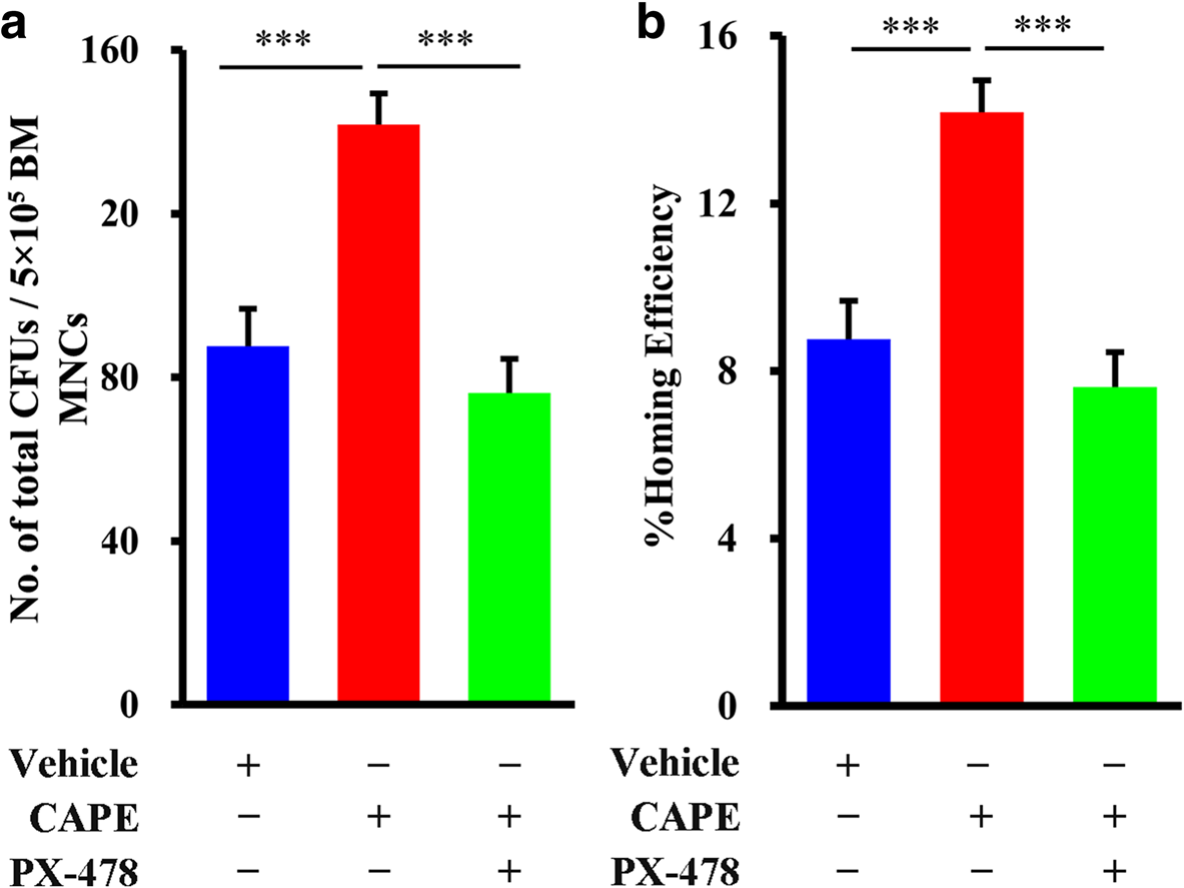
HIF-1α inhibitor PX-478 blocked CAPE-enhanced HSPC homing. **a** Total CFU number generated from 5 × 10^5^ BM MNCs (*n* = 5). Lethally irradiated mice were injected intraperitoneally with vehicle or 3.0 mg/kg CAPE from day –1 to day +1, and received BMT on day +1. For some mice, 5 mg/kg PX-478 was administered just after CAPE injection. Twenty hours after BMT, BM MNCs were harvested and cultured for CFU assays. Experiment repeated three times. **b** Homing efficiency calculated by comparing the homed CFU numbers with the initially injected CFU numbers. Data presented as mean ± SD. ***P < 0.001, CAPE vs Vehicle, CAPE vs CAPE + PX-478. BM bone marrow, CAPE caffeic acid phenethyl ester, CFU colony-forming unit, MNC mononuclear cell

Several studies have suggested that VEGF and SDF-1α are target genes of HIF-1α [26, 31, 32]. Based on these data, we hypothesized that HIF-1α, which can further regulate the expression of VEGF and SDF-1α, is the key target gene of CAPE. We then performed a homing experiment to assess whether blockade of HIF-1α expression using an inhibitor attenuated the CAPE-enhanced HSPC homing effect. Lethally irradiated mice were injected intraperitoneally with vehicle or 3.0 mg/kg CAPE at the same schedule as shown in Fig. 2a, and they received BMT. For some mice, 5 mg/kg PX-478 was administered just after daily CAPE injection. Twenty hours after transplantation, BM MNCs were harvested and cultured for CFU assays. The total number of CFUs in the CAPE group was greater than that in the vehicle group, and this difference was remarkably attenuated by injection with the HIF-1α inhibitor PX-478 (vehicle vs CAPE vs CAPE and PX-478, 87.6 ± 9.16 vs 141.7 ± 7.61 vs 76.1 ± 8.34 CFU number per 5 × 10^5^ BM MNCs) (Fig. 6a). The CAPE-mediated increase in HSPC homing efficiency was significantly inhibited by PX-478 (Fig. 6b). These results indicated that CAPE-enhanced HSPC homing to the BM was primarily dependent on regulation of HIF-1α.

## Discussion

HSCT is an effective and life-saving therapeutic strategy for patients with malignant and non-malignant disorders. Infused HSPCs are required to undergo several crucial processes to complete repopulation, including efficient homing to the BM from the circulating blood, engraftment in the BM and regeneration of all blood cell types. There are at least two ways to enhance homing of infused HSPCs to the BM, especially for limited numbers of HSPCs from CB. In-vitro manipulation of HSPCs to increase their number is an ex-vivo approach to increase HSPC homing and engraftment to the BM [33]. Improving chemotactic or adherent ability of HSPCs to the BM by ex-vivo treatment of HSPCs is also proved to be an effective strategy to enhance HSPC seeding to the BM. Some efforts include short-term exposure of HSPCs to PGE2, and short-term treatment of HSPCs with hyperthermia or hyperbaric oxygen [10, 34, 35]. To enhance HSPC homing and engraftment in the BM, another strategy is to give drugs to patients receiving HSPC transplantation [36], which is a relatively simple method that avoids the problems caused by ex-vivo manipulation. The drug sitagliptin has been reported to be used in patients for improving homing and engraftment of CB stem cells in clinical trials, which functions *in vivo* mainly via regulating the chemotactic activity of the transfused HSPCs [37]. Given that several chemotactic factors in the BM microenvironment have been proved to be involved in the retention of HSPCs, using drugs to improve the BM niche of patients is becoming a novel strategy [38, 39]. However, development of this kind of drug is still a challenge. Here, we found that CAPE, a natural compound extracted from honeybee hives, showed the potential to become this kind of candidate drug mainly via regulating the BM microenvironment.

CAPE is found in many plants and can also be synthesized by reacting caffeic acid with phenethyl alcohols [40, 41]. The various effects of CAPE are related to the dose, target cell type and disease model. In our study, we found that treatment of the recipients with CAPE enhanced HSPC homing and engraftment in the BM. By applying survival rate experiments in lethally irradiated mice with limited BM cell transplantation and CAPE treatment, we confirmed that CAPE injection to lethally irradiated recipients had a notably positive role in improving the survival rate and haematopoietic repopulation in mice receiving BMT. The dose and frequency of CAPE injection were different from that used in other disease models. For HSPC homing and engraftment experiments, a frequently used mouse model—that is, lethally irradiation with BMT [10, 30]—was chosen to evaluate the effect of CAPE. An optimal schedule for administration of CAPE at 3.0 mg/kg to the recipients from day –1 to +1 was further confirmed to be effective in significantly improving HSPC homing and subsequent short-term and long-term engraftment.

**Fig. 7:**
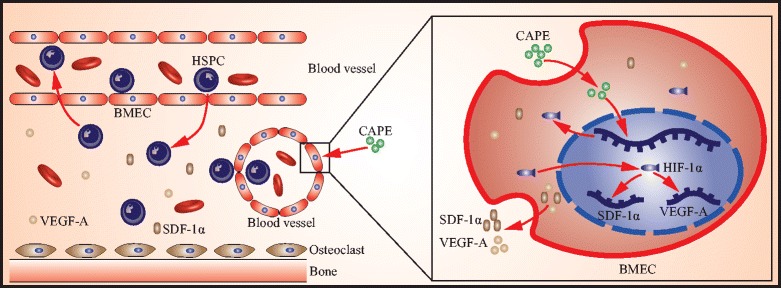
Graphic representation of the regulation mechanism of CAPE in the improvement of HSPC homing. Left: process of HSPC homing and engraftment in the BM, including HSPC rolling in vessels, migrating across BMECs and lodging in the BM niche. Right: CAPE induces expression of HIF-1α protein, activating downstream SDF-1α and VEGF-A gene expression in BMECs. Thus, CAPE creates a better chemotactic and survival environment for transplanted HSPCs and promotes HSPC homing and engraftment. Picture drawn by Dr Hailiang Li. BMEC bone marrow endothelial cell, CAPE caffeic acid phenethyl ester, HIF-1α hypoxia inducible factor-1α, HSPC haematopoietic stem/progenitor cell, SDF-1α stromal cell-derived factor 1α, VEGF vascular endothelium growth factor

Increasing evidence has indicated that different mechanisms are involved in the various functions of CAPE, including induction of HO-1 expression, activation of the ERK1/2-CREB signalling cascade and inhibition of NF-κB signals in different cell contexts and different disease models [42–45]. We found that CAPE upregulated the HIF-1α and SDF-1α gene and protein expression in BMECs, which further supports the hypothesis that CAPE has the ability to improve haematopoietic cell homing by regulating the BM niche (Fig. 7). SDF-1α is primarily expressed and secreted by BM niche cells, such as endothelial cells, stromal cells and osteoblasts. The SDF-1α level in the BM niche is a critical determinant for efficient HSPC recruitment and homing [4, 10, 46]. CAPE-enhanced SDF-1α immunostaining in BM microvessels suggested that the target cells of CAPE in irradiated BM were BMECs. BM mesenchymal-like stromal cells were not the target cells of CAPE, as evidenced by their non-responsiveness to CAPE. In addition to SDF-1α, VEGF-A, which functions as a survival factor for endothelial cells and haematopoietic stem cells, was also increased in the BM niche. Taken together, the increased SDF-1α and VEGF-A concentration in the BM niche created a better chemotactic and survival environment for transplanted HSPCs and led to increased HSPC homing to the damaged BM. Several studies have indicated that both SDF-1α and VEGF are downstream target genes of the transcriptional factor HIF-1α [31, 32]. In our experiments, we found that CAPE upregulated the expression of HIF-1α. By performing a HIF-1α inhibitor blocking experiment, we further confirmed that HIF-1α was a key point for inhibiting CAPE-induced HSPC homing. In future, more work needs to be done to clarify the mechanism of CAPE in activating HIF-1α transcription and extend these findings. Furthermore, comparison of the effect of CAPE derivatives with that of CAPE might be helpful to find more efficient candidate drugs for improvement of HSPC homing and engraftment in the BM.

## Conclusions

Our data are the first to show that CAPE administration to irradiated BMT recipients promotes recruitment and homing of HSPCs to the BM niche and subsequently improves the short-term and long-term engraftment of HSPCs in a BM transplantation model. The mechanism appears to involve activation of HIF-1α and downstream SDF-1α and VEGF-A gene expression in BMECs and creating a better BM niche for HSPC homing. Thus, we present a potent application for CAPE in improving homing and engraftment outcomes in patients receiving HSCT.

## Additional files

Additional file 1: Table S1. Presenting primers for Q-PCR. (DOCX 11 kb)

Additional file 2: Figure S1. Showing proteins detected in BM supernatants from lethally irradiated mice treated with three doses of CAPE or vehicle 20 h after transplantation, *n* = 3. Femurs of three mice per group were flushed using 5 ml PBS. Pooled supernatants per group were collected after centrifugation, freeze-dried and resuspended in 120 μl PBS. Cytokine array performed using the RayBio® Mouse Cytokine Antibody Array G-Series 3 (RayBiotech) following the manufacturer’s instructions. (TIF 1214 kb)

Additional file 3: Supplementary methods for isolation and characterization of BM stromal cells. (DOCX 10 kb)

Additional file 4: Figure S2. Showing expression of HIF-1α, VEGF-A and SDF-1α in primary mouse BMSCs. a, b Gene and protein expression levels of HIF-1α, VEGF-A and SDF-1α in primary mouse BMSCs. BMSCs were incubated with CAPE at different concentrations (0, 0.1, 1.0 μg/ml) respectively for 24 h. All gene expression data normalized by the housekeeping gene, β-ACTIN. Every test was repeated three times. (TIF 323 kb)

## Abbreviations

BFU-E: Erythrocyte burst-forming unit
BM: Bone marrow
BMEC: Bone marrow endothelial cell
BMSC: Bone marrow stromal cell
BMT: Bone marrow transplantation
CAPE: Caffeic acid phenethyl ester
CB: Cord blood
CFU: Colony-forming unit
CFU-G: Granulocyte colony-forming units
CFU-GEMM: Granulocyte–erythroblast–macrophage–megakaryocyte colony-forming units
CFU-GM: Granulocyte–macrophage colony-forming units
CFU-Meg: Megakaryocyte colony-forming units
DMSO: Dimethyl sulphoxide
FVS510: Fixable Viability Stain 510
HIF-1α: Hypoxia inducible factor-1α
HO-1: Haeme oxygenase-1
HSCT: Haematopoietic stem cell transplantation
HSPC: Haematopoietic stem/progenitor cell
Lin: Lineage
LSK: Lin^–^Sca-1^+^c-Kit^+^
MNC: Mononuclear cell
PB: Peripheral blood
PGE2: Prostaglandin E2
SCF: Stem cell factor
SDF-1α: Stromal cell-derived factor 1α
SSC: Side scatter
TBI: Total body irradiation
VEGF: Vascular endothelium growth factor
