# Supplementary material for: Caffeic acid phenethyl ester promotes haematopoietic stem/progenitor cell homing and engraftment
Source: Stem Cell Res Ther. 2017 Nov 7;8:255. doi: 10.1186/s13287-017-0708-x (PMC5678809; doi:10.1186/s13287-017-0708-x)
Supplement: Supplementary file 1 — Presenting primers for Q-PCR. (DOCX 11 kb) [file 13287_2017_708_MOESM1_ESM.docx]

**Additional file 1: Table. S1** Primers for Q-PCR

|  | Forward primers | Reverse primers |
| --- | --- | --- |
| β-ACTIN | GTGACGTTGACATCCGTAAAGA | GCCGGACTCATCGTACTCC |
| HIF-1α | ACCTTCATCGGAAACTCCAAAG | ACTGTTAGGCTCAGGTGAACT |
| SDF-1α | TGCATCAGTGACGGTAAACCA | CACAGTTTGGAGTGTTGAGGAT |
| VEGF-A | GCACATAGAGAGAATGAGCTTCC | CTCCGCTCTGAACAAGGCT |
