# Supplementary figures and images for: Caffeic acid phenethyl ester promotes haematopoietic stem/progenitor cell homing and engraftment
Source: Stem Cell Res Ther. 2017 Nov 7;8:255. doi: 10.1186/s13287-017-0708-x (PMC5678809; doi:10.1186/s13287-017-0708-x)

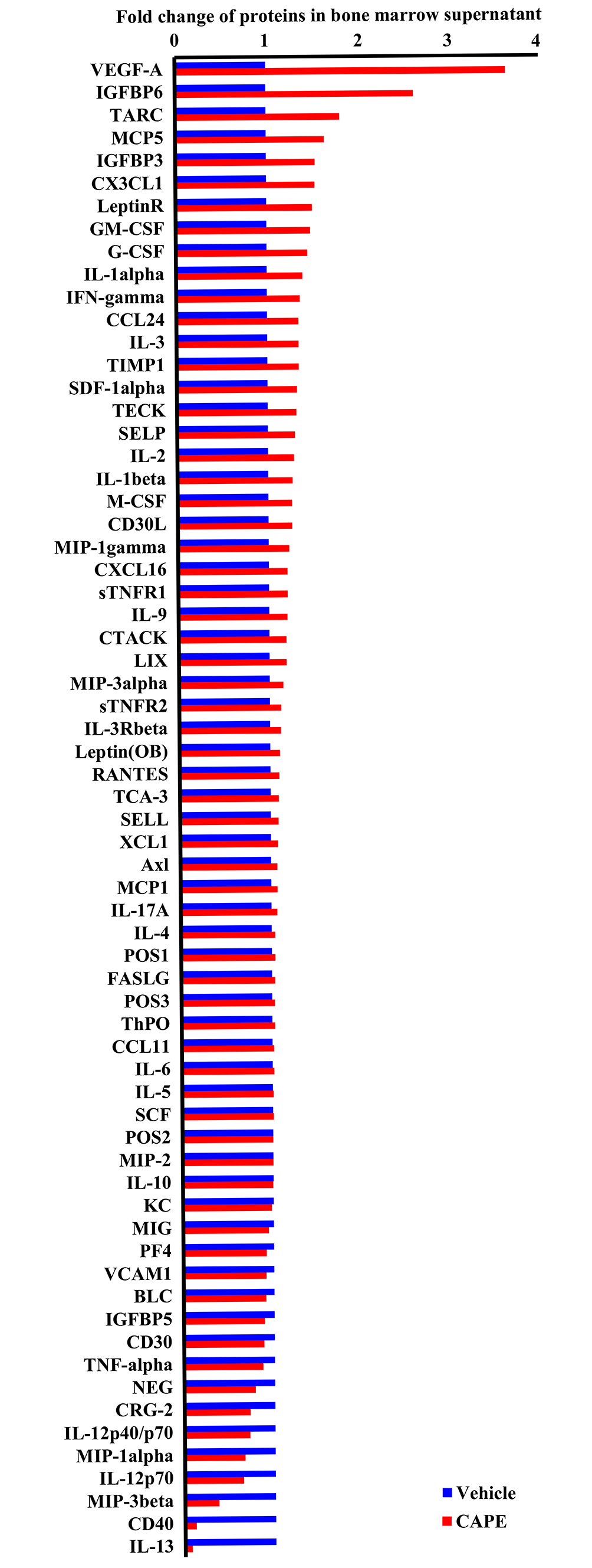

Supplement: Supplementary file 2 — Showing proteins detected in BM supernatants from lethally irradiated mice treated with three doses of CAPE or vehicle 20 h after transplantation, n = 3. Femurs of three mice per group were flushed using 5 ml PBS. Pooled supernatants per group were collected after centrifugation, freeze-dried and resuspended in 120 μl PBS. Cytokine array performed using the RayBio® Mouse Cytokine Antibody Array G-Series 3 (RayBiotech) following the manufacturer’s instructions. (TIF 1214 kb) [file 13287_2017_708_MOESM2_ESM.tif]

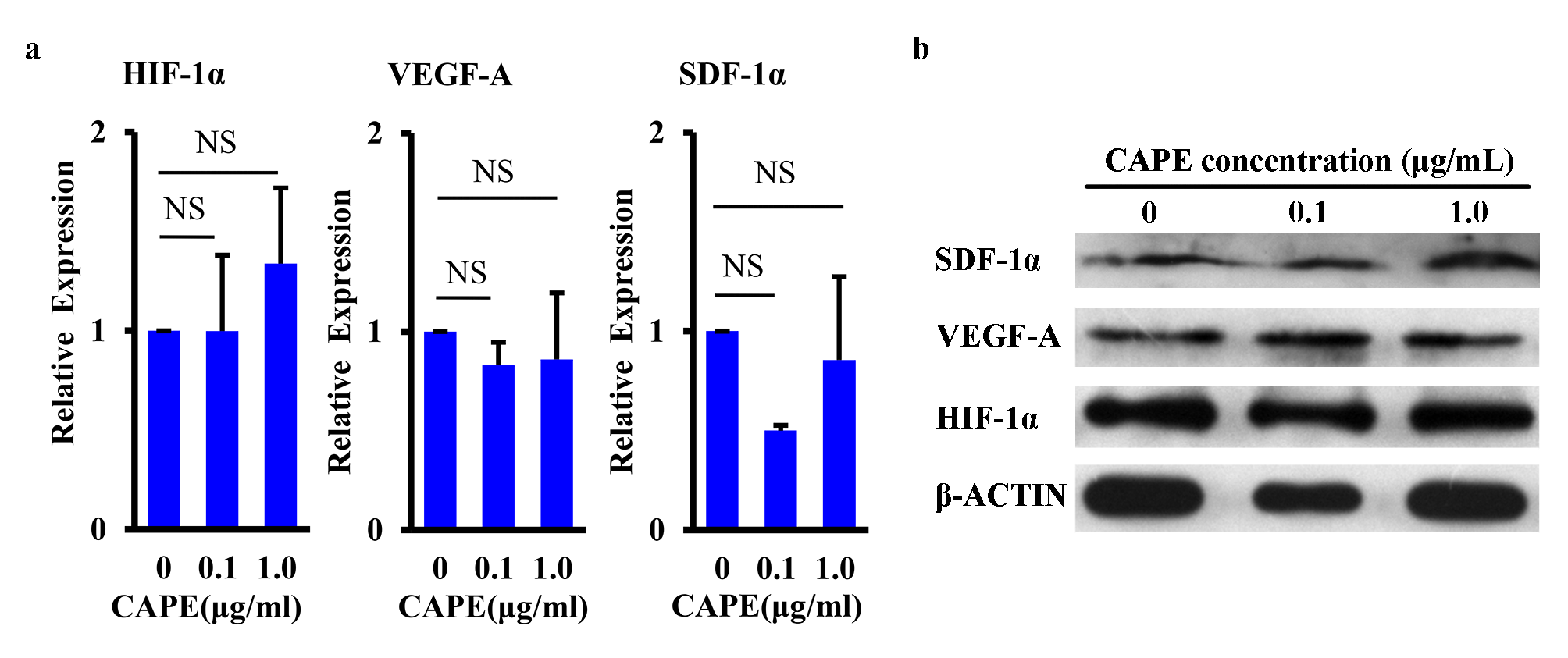

Supplement: Supplementary file 4 — Showing expression of HIF-1α, VEGF-A and SDF-1α in primary mouse BMSCs. a, b Gene and protein expression levels of HIF-1α, VEGF-A and SDF-1α in primary mouse BMSCs. BMSCs were incubated with CAPE at different concentrations (0, 0.1, 1.0 μg/ml) respectively for 24 h. All gene expression data normalized by the housekeeping gene, β-ACTIN. Every test was repeated three times. (TIF 323 kb) [file 13287_2017_708_MOESM4_ESM.tif]
