# Supplementary material for: Caffeic acid phenethyl ester promotes haematopoietic stem/progenitor cell homing and engraftment
Source: Stem Cell Res Ther. 2017 Nov 7;8:255. doi: 10.1186/s13287-017-0708-x (PMC5678809; doi:10.1186/s13287-017-0708-x)
Supplement: Supplementary file 3 — Supplementary methods for isolation and characterization of BM stromal cells. (DOCX 10 kb) [file 13287_2017_708_MOESM3_ESM.docx]

**Additional file 3: Isolation and characterization of BM stromal cells:** Briefly, BM cells (BMSCs) from tibias and femurs of C57BL/6 mice were flushed with Alpha MEM medium (Gibco, Carlsbad, CA) containing 2% fetal bovine serum (FBS, Gibco, Carlsbad, CA). All cells were seeded into T-25 cm^2^ flasks containing Alpha MEM and 20% FBS. The BM stromal cells cultures grew at 37°C in 5% CO_2_. The medium was removed after 3 days by washing with PBS followed by media changes every 3-4 days. As the cells grew to 80% confluence, the BM stromal cells were recovered using 0.25% Trypsin–0.02% EDTA and replated at 1:3. Primary BM stromal cells exhibited their characteristic spindle-shaped morphology and gave rise to small colonies.
